# High spatial and temporal resolution retrospective cine cardiovascular magnetic resonance from shortened free breathing real-time acquisitions

**DOI:** 10.1186/1532-429X-15-102

**Published:** 2013-11-14

**Authors:** Hui Xue, Peter Kellman, Gina LaRocca, Andrew E Arai, Michael S Hansen

**Affiliations:** 1 National Heart, Lung and Blood Institute, National Institutes of Health, 10 Center Drive, Bethesda, MD 20814, USA

**Keywords:** Retrospective reconstruction, Myocardial function, Motion correction, Real-time imaging, Cardiac MRI

## Abstract

**Background:**

Cine cardiovascular magnetic resonance (CMR) is challenging in patients who cannot perform repeated breath holds. Real-time, free-breathing acquisition is an alternative, but image quality is typically inferior. There is a clinical need for techniques that achieve similar image quality to the segmented cine using a free breathing acquisition. Previously, high quality retrospectively gated cine images have been reconstructed from real-time acquisitions using parallel imaging and motion correction. These methods had limited clinical applicability due to lengthy acquisitions and volumetric measurements obtained with such methods have not previously been evaluated systematically.

**Methods:**

This study introduces a new retrospective reconstruction scheme for real-time cine imaging which aims to shorten the required acquisition. A real-time acquisition of 16-20s per acquired slice was inputted into a retrospective cine reconstruction algorithm, which employed non-rigid registration to remove respiratory motion and SPIRiT non-linear reconstruction with temporal regularization to fill in missing data. The algorithm was used to reconstruct cine loops with high spatial (1.3-1.8 × 1.8-2.1 mm^2^) and temporal resolution (retrospectively gated, 30 cardiac phases, temporal resolution 34.3 ± 9.1 ms). Validation was performed in 15 healthy volunteers using two different acquisition resolutions (256 × 144/192 × 128 matrix sizes). For each subject, 9 to 12 short axis and 3 long axis slices were imaged with both segmented and real-time acquisitions. The retrospectively reconstructed real-time cine images were compared to a traditional segmented breath-held acquisition in terms of image quality scores. Image quality scoring was performed by two experts using a scale between 1 and 5 (poor to good). For every subject, LAX and three SAX slices were selected and reviewed in the random order. The reviewers were blinded to the reconstruction approach and acquisition protocols and scores were given to segmented and retrospective cine series. Volumetric measurements of cardiac function were also compared by manually tracing the myocardium for segmented and retrospective cines.

**Results:**

Mean image quality scores were similar for short axis and long axis views for both tested resolutions. Short axis scores were 4.52/4.31 (high/low matrix sizes) for breath-hold vs. 4.54/4.56 for real-time (paired *t*-test, P = 0.756/0.011). Long axis scores were 4.09/4.37 vs. 3.99/4.29 (P = 0.475/0.463). Mean ejection fraction was 60.8/61.4 for breath-held acquisitions vs. 60.3/60.3 for real-time acquisitions (P = 0.439/0.093). No significant differences were seen in end-diastolic volume (P = 0.460/0.268) but there was a trend towards a small overestimation of end-systolic volume of 2.0/2.5 ml, which did not reach statistical significance (P = 0.052/0.083).

**Conclusions:**

Real-time free breathing CMR can be used to obtain high quality retrospectively gated cine images in 16-20s per slice. Volumetric measurements and image quality scores were similar in images from breath-held segmented and free breathing, real-time acquisitions. Further speedup of image reconstruction is still needed.

## Background

Cine cardiovascular magnetic resonance (CMR) is an accepted reference standard for assessing myocardial morphology and function [1]. Currently, the available clinical techniques rely on breath-held, segmented acquisitions to fill the *k*-space over multiple heartbeats using retrospective ECG gating. With this approach only one or two slices are acquired per breath-hold thus requiring multiple breath-holds to cover the ventricles for functional evaluation. Although this strategy is reliable in many clinical patients, it leads to a lengthy exam. The reliance on breath-holding is also problematic in a patient population where shortness of breath is prevalent. For subjects who cannot hold their breath, the segmented cine imaging falls short. Even in patients who can hold their breath at the beginning of the study, they often fail to maintain consistent breath-hold compliance throughout the study.

To overcome these shortcomings of segmented breath-held acquisitions, real-time imaging can be utilized without the need for breath-holding or ECG triggering. Real-time cine CMR [2-4] utilizes accelerated imaging and single-shot acquisitions to acquire snapshot images of the heart fast enough to freeze the cardiac and respiratory motion. Therefore, it is less susceptible to imperfect breath-holding and arrhythmias. In spite of these advantages, real-time CMR often suffers from reduced image quality due to compromised spatial and temporal resolution [5] when compared to a successful segmented, breath-held cine acquisition. Clinically, real-time techniques are most frequently used for arrhythmia cases and segmented cine with multiple averages may be used for patients who cannot hold their breath.

Different approaches have been proposed to improve image quality of real-time cine CMR. The majority relies on imaging with high undersampling factors to achieve better spatial/temporal resolution. These techniques often utilize temporal or spatiotemporal redundancy of cine CMR. In some techniques, parallel imaging calibration is obtained from a time average of the data [6-8], and in others, signal redundancy in the spatial-temporal frequency domain is exploited [9]. Image domain regularization also has been used to reduce image artifacts [10-13]. While improved image quality has been reported, the achievable spatial/temporal resolution still does not match the segmented cine acquisition. Also, the reconstructed real-time images are often not retrospectively gated to cover the entire cardiac cycle, which poses some practical difficulties when extracting quantitative measures of myocardial function. A comprehensive review of temporal acceleration techniques for dynamic imaging has been published [14].

Real-time CMR can, however, be used to acquire *k*-space data over multiple heartbeats during free breathing. This opens the possibility of combining these data to obtain retrospectively gated reconstructions based on the recorded ECG signal and motion correction to compensate for the respiratory motion. This observation has led to a set of approaches, which improve real-time cine CMR [15-17]. These techniques separate themselves from more traditional real-time imaging techniques by combining *k*-space data across multiple cardiac cycles and by producing a retrospectively gated image series covering the entire cardiac cycle.

The first method of this kind produced retrospective reconstruction of real-time images by averaging images from multiple cardiac cycles to improve the SNR [15]. The real-time images were first reconstructed using parallel imaging (e.g. TGRAPPA [7]) and then corrected for the respiratory motion with non-rigid registration. An image based respiratory navigator signal was estimated from the real-time images and used to discard images with excessive respiratory motion excursion. The main drawback of this method was that the underlying temporal resolution of reconstructed images was unchanged. This issue was addressed by extending the image domain averaging to *k*-space binning [16]. In this improved approach, the real-time images acquired at matching cardiac cycles were transformed back to *k*-space. Trigger times for every *k*-space line were determined from the recorded ECG signal. A *k*-space binning was then performed by re-assembling readout lines based on the real cardiac cycle acquisition time. For a cardiac phase, only those lines falling into its temporal window were included for its subsequent reconstruction to image space using a simple Fourier transform. Although this technique was successfully employed to improve the temporal resolution, a major limitation of this *k*-space binning based approach was that a rather lengthy acquisition (e.g. 60s per slice in [15]) was needed to ensure the complete filling of binned *k*-space. This drawback was partially overcome by incorporating the parallel imaging into the reconstruction of the binned *k*-space of every cardiac phase [17]. This last method incorporated a multi-shot motion correction formula [18] into a general, iterative SENSE reconstruction. Good image quality was reported using 30s of real-time data.

In the current study we propose to further shorten the required acquisition duration of the retrospective real-time cine acquisition by employing a non-linear reconstruction step. This enables a shortened acquisition of 16-20s and generates image quality comparable to the segmented cine with good spatial (1.3-1.8 × 1.8-2.1 mm^2^) and temporal (retrospectively gated, 30 cardiac phases) resolution. Additionally, previous studies [16,17] showed results for a single short axis cine slice. In the current study, myocardial function is evaluated for the entire left ventricle with multiple short axis and long-axis views acquired. A quantitative comparison is performed between real-time retrospective reconstruction and corresponding breath-held, segmented cine acquisitions with identical slice prescriptions.

## Methods

### Retrospective reconstruction

Retrospective reconstruction of real-time cardiac cine was performed on *k*-space data acquired during free breathing over multiple heartbeats. To achieve sufficient spatial resolution (e.g. 256 × 144 matrix, pixel size ~1.5 × 1.5 mm^2^) matching the segmented acquisition, the real-time image series had relatively low temporal resolution (~80-110 ms) and were subsequently rebinned to form a series of high temporal resolution images. To perform the *k*-space data combination across heartbeats, the acquired data from different heartbeats are required to be sufficiently similar (respiratory phase) and heart beats which were significantly shorter or longer than average (in this study, accepted heartbeats were required to have an RR interval within ±50% of mean RR) were rejected from data processing. Non-rigid registration was applied to further correct residual respiratory motion. The motion corrected, respiratory gated, real-time data was then converted to *k*-space and binned to desired cardiac phases based on the recorded ECG signal.

**Figure 1:**
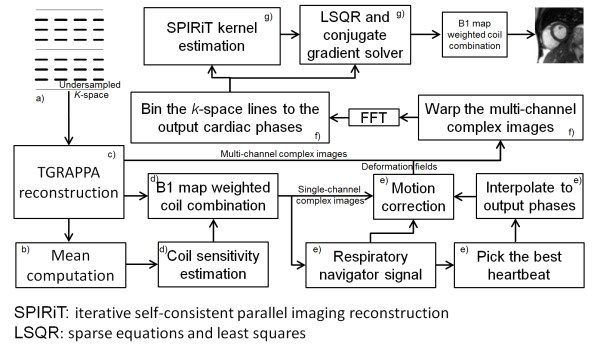
Schematic diagram of proposed reconstruction scheme.

Figure 1 illustrates the entire process of proposed retrospective reconstruction. ***a)*** Undersampled k-space data were acquired with parallel imaging reduction factor *R* = 4 and a time-interleaved (TGRAPPA/TSENSE) sampling pattern. ***b)*** To reconstruct the underlying real-time cine images, the auto-calibration signal (ACS) data was obtained by averaging all undersampled *k*-space frames. This is equivalent to a sliding window moving average with the window including the entire series. This “average-all” strategy speeds up the TGRAPPA reconstruction by only computing the GRAPPA kernel once and applying it to all frames. ***c)*** The standard GRAPPA reconstruction was performed on every undersampled *k*-space frame. ***d)*** After the coil-by-coil GRAPPA reconstruction, phased array combination coefficients were estimated from the mean complex image of every channel. A rank-1 eigen-analysis based coil sensitivity estimation method [19] was used. The coil combination was performed by multiplying the complex conjugates of the estimated combination coefficients with the complex images on every channel and summing over all the channels. In this way, the acquired raw real-time images were reconstructed with low temporal resolution and high spatial resolution. ***e)*** An image-based respiratory signal was computed from the real-time cine images, as previously proposed in [15] where the prominent respiratory motion direction is adapted to the slice orientation. This signal was used to discard data with extreme respiratory positions. It was also used to pick a heartbeat at end-expiration. Images from this heartbeat served as the reference for image registration to correct in-plane respiratory motion [16]. The acceptance window was set to be 50% of the maximal respiratory motion. ***f)*** After correcting the respiratory motion using non-rigid registration, the complex real-time images inside the respiratory acceptance window were transformed back to *k*-space. Every *k*-space line was assigned to a cardiac phase bin based on the ECG time stamp. In our experiments, 30 output cardiac phases were prescribed, leading to a temporal resolution of ~33 ms at a nominal heart rate of 60 bpm. If higher temporal resolution is required, more output phases can be generated by narrowing the cardiac binning window, albeit at the expense of a longer acquisition time. Although the real-time cine data were acquired without explicit ECG triggering, all readout lines were labeled with acquisition time and ECG trigger time. These time-stamps were used to assign every readout line into correct cardiac phase. A simple retrospective arrhythmia rejection scheme was also implemented. The mean RR interval was first computed over all acquired heartbeats. The *k*-space data was accepted for binning only if it was from heartbeats with RR interval length within ±50% of mean RR. This threshold was sufficient for the current study, but may need to be reduced for patients with strong arrhythmia. ***g)*** With the shortened acquisition, the binned *k*-space is often incomplete, showing an irregular undersampling pattern. This degrades the image quality of a simple FFT reconstruction. Therefore, to fill these holes and stabilize the reconstruction, a non-linear SPIRiT reconstruction [20,21] with spatio-temporal regularization was applied to compute the retro-gated *k*-space and resulting magnitude images. This is a key step in enabling the shortened acquisition while maintaining good spatial and temporal resolution comparable to the segmented cine acquisition.

### SPIRiT reconstruction with spatial-temporal regularization

Assume the binned 2D + T *k*-space ***x*** consists of filled points ***a*** and missing points ***m***, then:

(1) $$x=D^{T}a+D_{c}^{T}m$$

Here ***x*** is an ***N*** **× 1** vector containing *k*-space points for all phase encoding lines of all ***P*** cardiac phases and all channels; ***α*** is the ***A*** **× 1** vector of filled points, and ***m*** is the ***M*** **× 1** vector for missing points. ***D*** is the ***A*** **×** ***N*** sampling matrix which selects the filled points and ***D***_*c*_ is the ***M*** **×** ***N*** sampling matrix which selects the missing points. The operator ***D***^***T***^ is used to put the filled points back in the right location in a ***N*** **×** 1 k-space vector and all locations corresponding to missing points are zero. The operator $D_{C}^{T}$ can be similarly computed for the missing points.

The SPIRiT reconstruction proposes to fit every *k*-space point using all of its close spatial neighbors [20], no matter whether those neighboring points are acquired or missing. In this way, filled points and missing points are mixed together to define a large set of coupled linear equations. The SPIRiT equation uses a self-consistent *k*-space operator: suppose ***x*** is the optimal *k*-space to be reconstructed, it should be a stationary point for the SPIRiT kernel ***G*** [20]:

(2) x=Gx

By separating acquired and missing points from ***x***:

(3) $$\begin{array}{c} \left(G‒I\right)\left(D^{T}a+D_{c}^{T}m\right)=0\\ \left(G‒I\right)D_{c}^{T}m=\left(G‒I\right)D^{T}a \end{array}$$

Here ***G*** is the SPIRiT kernel matrix which is computed from a fully sampled set of auto calibration data [20]. Although SPIRiT equation 3 appears as a linear equation, it is not trivial to invert it directly, because of the dimension of ***G*** and because both missing and filled points are coupled. As a consequence, equation 3 is practically solved with iterative methods.

The 2D + T *k*-space ***x*** contains the samples from all cardiac phases. The SPIRiT kernel ***G*** is thus a block diagonal matrix, consisting of *P* sub-blocks for every 2D cardiac phase.

The linear SPIRiT equation 3 can be extended by adding image domain regularization terms [20]. In this study, equation 3 is extended and reformulated as an optimization problem:

(4) $$\mathrm{argmi}n_{x}\left\{∥\left(G‒1\right)x{∥}_{2}+\lambda ∥\Psi C^{H}F^{H}x{∥}_{1}+∥\text{Dx}‒a{∥}_{2}\right\}$$

***ψ*** is the 3D redundant Harr wavelet transformation matrix and applied to the complex 2D + T cardiac images. This operator computes the wavelet coefficients along both spatial and temporal direction. Thus this non-linear reconstruction step utilizes both spatial and temporal regularization. ***F*** is the Fourier transform matrix, converting every cardiac phase images to *k*-space. ***C*** is the coil sensitivity. ***C***^***H***^ ***x*** represents the coil combination, converting the multi-channel complex images to the coil combined single-channel images for all cardiac phases. Note the data fidelity term is always weighted by 1.0 in our experiments. Thus only one tunable parameter ***λ*** is included in the equation above.

To assemble the ACS data for SPIRiT kernel estimation, the warped *k*-space after respiratory gating was averaged at every output cardiac phase. Instead of using the raw *k*-space mean as the ACS, the SPIRiT kernels were estimated on the warped mean *k*-space. This strategy minimizes mismatches possibly caused by *k*-space warping and respiratory motion between the binned *k*-space and ACS data. The SPIRiT kernel size used was 7 × 7 and the regularization weight ***λ*** was set to be 0.001 for all experiments. This parameter setting was empirically selected to balance the good SNR and temporal fidelity of reconstruction. To achieve faster convergence, the linear SPIRiT equation 3 was first solved using the LSQR algorithm [22]. After the convergence of the LSQR solver, the non-linear conjugate gradient optimization was initialized with the output of to solve the nonlinear problem (equation 4).

### Motion correction

Non-rigid deformation fields are needed in the estimation of image-based respiratory navigator and for warping the real-time complex cine images before the *k*-space binning. To estimate the deformation field, a non-rigid image registration algorithm [17] was applied. The registration algorithm employed regularization on the deformation field to constrain its smoothness. To speed up the convergence and avoid local minima, a multi-scale approach was used (5 levels in all experiments). Maximal 100 iterations were performed on every scale until the convergence. After computing the deformation fields, the complex real-time images were warped using a fifth order BSpline interpolator and then converted back to *k*-space for binning.

### Imaging experiments

Imaging experiments were performed on a 3T clinical MRI system (MAGNETOM Skyra, Siemens AG Healthcare Sector, Erlangen, Germany) equipped with 64 receive channels. The study was approved by the local Institutional Review Board, and all subjects gave written informed consent.

Fifteen (N = 15) healthy volunteers (10 men, 5 women; mean age 32.9 ± 11.2 years) were recruited for the study and imaged using steady state free precession sequences. To validate the proposed approach for cardiac function evaluation, both breath-held, segmented cine and free-breathing, real-time imaging was performed. Every subject was scanned with two matrix sizes: 256 × 144 and 192 × 128. For every matrix size, 8–12 short axis (SAX) slices were acquired to cover the ventricles. Additionally, three long axis (LAX) views (four-chamber, two-chamber, and three-chamber views) were acquired. In total, 644 SAX and 180 LAX image series were acquired with both segmented and real-time acquisitions at two matrix sizes. The same slice geometry was used for real-time and segmented acquisitions to facilitate quantitative and qualitative comparison.

Common sequence parameters for both breath-held and real-time cine were as follows: balanced SSFP readout, TR = 3.01/TE = 1.29 ms for 256 × 144 matrix and TR = 2.76/TE = 1.19 ms for 192 × 128 matrix, flip angle 40°, typical FOV 340-360 × 255-270 mm^2^, slice thickness 8 mm with a gap of 2 mm, bandwidth 930 Hz/pixel. An asymmetric echo acquisition (20%) was used. Segmented acquisitions were performed with retrospective ECG gating and 3-fold parallel imaging acceleration. Images were reconstructed with GRAPPA and 32 reference lines to produce 30 retro-gated cardiac phases covering the entire heartbeat. Typical breath-holding time of the segmented acquisition was 8-11s per slice. For the real-time acquisition, 4-fold parallel imaging acceleration with temporally interleaved acquisition was used.

Real-time acquisitions were performed with 4-fold acceleration and temporally interleaved sampling pattern. The acquired temporal resolution was ~90-120 ms and reconstructed to produce 30 phases to cover the entire cardiac cycle with the approach described above. A total of 20s real-time data was acquired for the matrix size of 256 × 144 and 16s of data for 192 × 128. As a result, the ventricles were covered for functional evaluation in 3–4 minutes of short-axis scan time during free-breathing, which significantly simplifies the scanning operation, compared to the “one slice per breath-hold” acquisition. One extra minute of scan time will be needed if long-axis views are going to be acquired.

### Inline reconstruction

**Figure 2:**
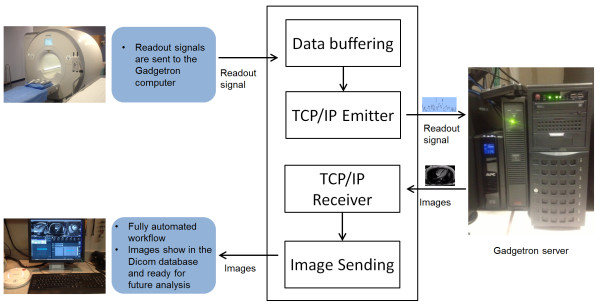
**An illustration of the Gadgetron based inline reconstruction of retrospective real-time cine imaging.** The reconstruction for a slice starts whenever all readout signal for this slice have been sent to the Gadgetron computer on the right. Once the reconstruction is complete, the images are sent back to the scanner host. In this setup, user has the flexibility to configure the external computer for more powerful hardware and software. The functionality of emitting data and receiving images were implemented on the vendor provided reconstruction computer attached to the magnet.

To deploy the proposed solution in the clinical setting, all processing steps were implemented using C++ on the Gadgetron [23] framework, which ran on a separate personal computer (four quad-core Intel Xeon E5-2670 2.60GHz processers, 20MB L3 cache, 256GB RAM). All processing is performed on the multi-core CPUs with the OpenMP based multi-threading. As illustrated in Figure 2, every readout data was sent to the Gadgetron computer in real-time. Once all data for one slice was fully acquired, the reconstruction of that slice was started, while the next slice was being scanned. The entire process was fully automated and all reconstructed images were sent back to the scanner host from the Gadgetron computer. The reconstruction processing took 80-120 s per slice and TGRAPPA real-time images were first reconstructed and sent back to scanner host for quick displaying before completion of the entire process. This inline reconstruction scheme was quite suitable for clinical usage, since the resulting images were directly stored in the clinical Dicom database and ready for further analysis.

### Experimental measurements

Retrospective reconstruction of real-time cine was compared to corresponding breath-hold segmented cine for short and long axis slices. The overall image quality for all subjects was assessed by two experts using a score between 1 and 5 (0.5 increment). For every subject, all LAX slices and three SAX slices (basal, medial and apex) were selected and reviewed for image quality. All reconstructed images were converted to movie files and viewed in the random order. The reviewers were blinded to the reconstruction approach and acquisition protocols of the images. For both matrix sizes, scores were given to segmented and retrospective cine series. The mean of two experts was taken as the final score for a series. A score of 1 indicated the worst quality and a score of 5 was the best. Specifically, a score of 5 (excellent) was given if a reconstruction had no noteworthy artifacts, good contrast between blood and myocardium and sharp boundaries. A score of 4 (good) was given for images with some insignificant artifacts, but good enough quality to determine cardiac function. A score of 3 (fair) was given to images with borderline image quality, which could still be used to evaluate global and regional function. A score of 2 (poor) meant insufficient image quality to evaluate regional function and the score of 1 was given (non-diagnostic) to images where it was impossible to evaluate global myocardial function.

Myocardial volumetric measurements were obtained by manually tracing (by a cardiologist) the end-diastolic (ED) and end-systolic (ES) myocardial boundary at both matrix sizes for all SAX slices. The end-diastolic volume (EDV), end-systolic volume (ESV), and ejection fraction (EF) were computed for segmented and real-time images. A paired *t*-test was used to compute the statistical significance between corresponding segmented and real-time measures. The one-way ANOVA was performed to compare the image quality scores.

## Results

**Figure 3:**
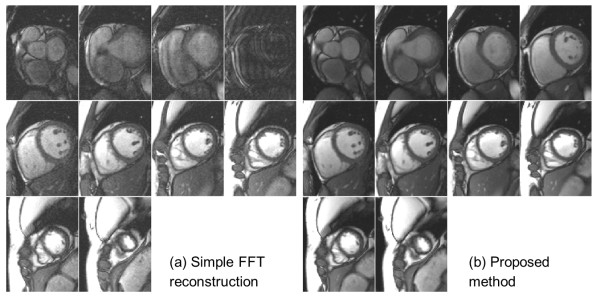
**Retrospective real-time cine images with shortened acquisition covering the ventricles.** The acquisition for this case is 256 × 144 matrix and 20 s per slice. A total of 10 SAX slices were acquired within 3.5 mins. FFT reconstruction **(a)** is not robust for the shortened acquisition, leading to degraded image quality, while proposed approach gives good image quality. The 4th image on the top of the left hand panel is an illustration of what happens when the central *k*-space lines are missing after binning.

**Figure 4:**
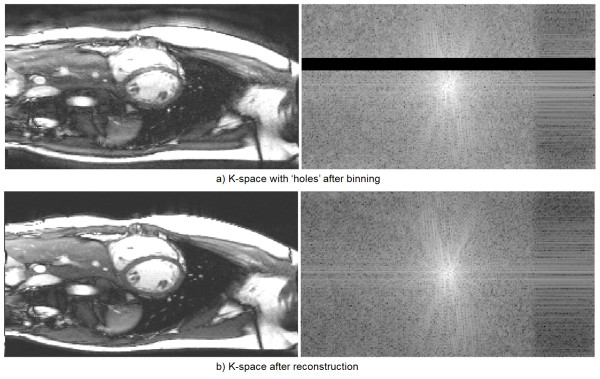
**Example of *k*-space of cine series with ‘holes’ after binning.** Missing lines in the binned *k*-space **(a)** degrades the image quality. After the reconstruction, missing data is filled and image quality is restored **(b)**. The right column shows the log magnitude of *k*-space. The 20% asymmetric echo portion can be seen on the right end of *k*-space.

Visual inspection of all reconstruction results confirmed the success of proposed method on all scans. Volumetric quantification was successfully achieved for all subjects. Figure 3 shows representative images covering the ventricles for a single subject (Additional file 1: Figure S3a.avi, Additional file 2: Figure S3b.avi). Compared to a simple FFT based reconstruction after *k*-space binning, the proposed non-linear approach filled the ‘holes’ in the binned *k*-space (Figure 4 and Additional file 3: Figure S4.avi) and improved the image quality of retrospective reconstruction. For both matrix sizes, at the tested acquisition duration, the FFT based reconstruction led to noticeable artifacts in all subjects. Although a reconstruction with fair image quality may be achieved for some slices, it was found to lack the robustness afforded by the non-linear reconstruction. The SPIRiT reconstruction step is necessary for the shortened acquisition.

**Figure 5:**
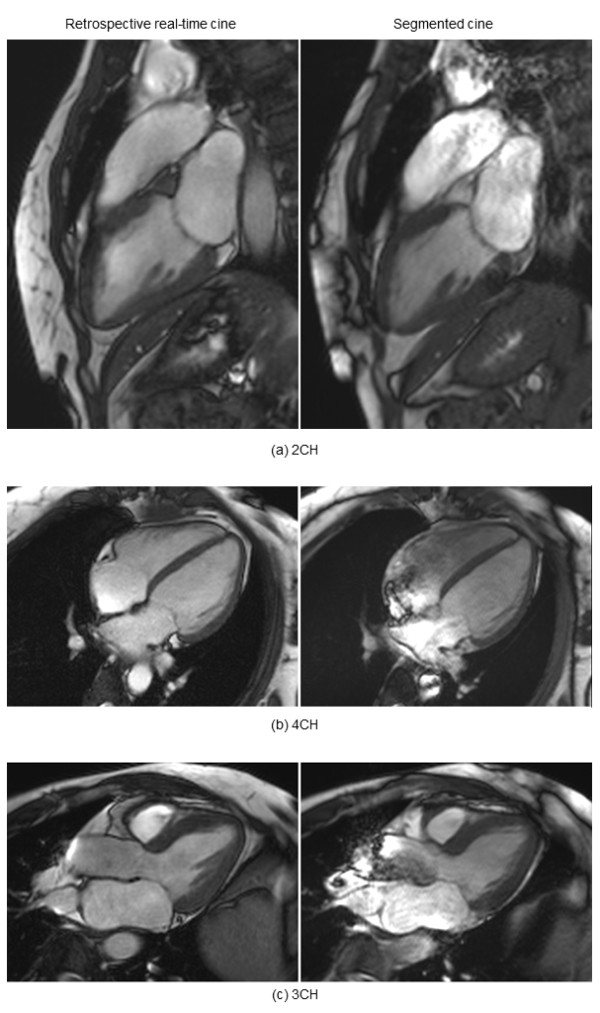
**Retrospective real-time cine for different long-axis views. (a)** the vertical long-axis or two-chamber view, **(b)** the four-chamber view, **(c)** the LV inflow/outflow or three-chamber view. The image quality of retrospective real-time cine on the left is comparable to the segmented breath-hold acquisition on the right. Both the real-time and segmented cine were acquired with 192 × 128 matrix. For the real-time cine, data from 16s of acquisition was used for reconstruction.

Figure 5 gives examples of retrospective reconstruction and segmented cine for variant long-axis views, demonstrating the quality of free-breathing retrospective reconstruction was comparable to the corresponding breath-held, segmented cine (Additional file 4: Figure S5a.avi, Additional file 5: Figure S5b.avi, Additional file 6: Figure S5c.avi).

**Figure 6:**
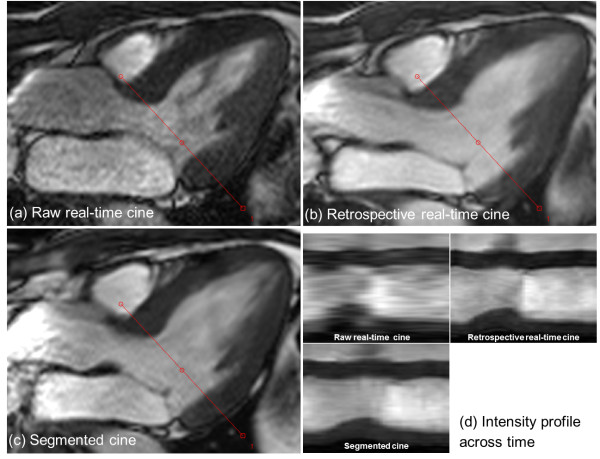
**Retrospective real-time cine with improved temporal resolution can enhance the visualization of valve.** In this 3CH slice, the mitral valve is well captured in both real-time reconstruction **(b)** and segmented cine **(c)**. The image quality of raw real-time cine **(a)** is much worse than the retrospective reconstruction. **(d)** shows the intensity profiles across time for the retrospective real-time and segmented cine. The intensity profile of raw real-time cine is generated by interpolating all images acquired in one cardiac cycle. The trigger time of raw real-time cine is 342 ms and for retro-gated reconstruction **(b** and **c)**, it is the 7th cardiac phase out of 30 with the approximated trigger time of 395 ms.

The mean RR interval for breath-held acquisition was 1039.0 ± 155.6 ms, reconstructed temporal resolution 34.6 ± 5.2 ms. For free-breathing acquisition, mean RR interval was 1028.7 ± 271.9 ms, reconstructed temporal resolution 34.3 ± 9.1ms. The mean breath-hold times for segmented acquisition were 10.9 ± 1.6 s for 256 × 144 matrix and 9.9 ± 1.4 s for 192 × 128 matrix. Compared to the 90-120 ms temporal resolution of acquired raw real-time cine, the improved temporal resolution is vital for valve visualization. Figure 6 shows a 3-chamber view with the mitral valve clearly visible in the retrospective images, while visualization of the valve was suboptimal on the raw real-time images due to insufficient SNR and low temporal resolution (Additional file 7: Figure S6.avi). The intensity profiles vs. time through the myocardium demonstrate that the retrospective reconstruction from real-time images and the segmented acquisition have comparable temporal characteristics. On the other hand, raw real-time images consistently gave inferior SNR and lower temporal resolution, which made them less attractive for clinical usage and volumetric quantification. While the inline reconstruction used in this study outputted both raw real-time and retrospective cine reconstructions for direct side-by-side comparison, we did not perform systematic image quality scoring between them. It was the consensus of the cardiologists that the raw real-time images were of inferior quality.

**Figure 7:**
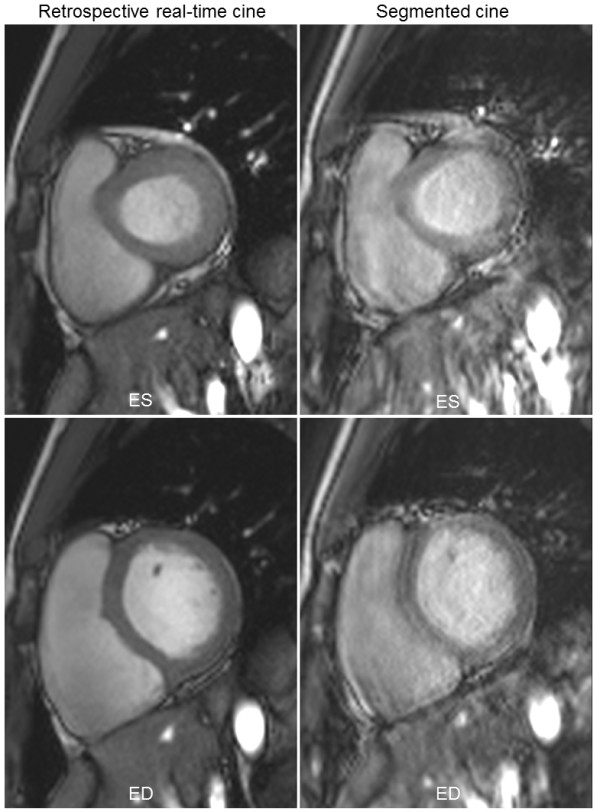
**An example of segmented cine degraded with imperfect breath-holding (right column), while the retrospective real-time cine (left column) gives good results.** Besides being tedious and time-consuming, the repeated breath-holding in the segmented cine also complicates the scanning procedure and reduces the robustness. Both ES (upper row) and ED phases (lower row) are shown.

The segmented cine can be degraded if the breath-holding is compromised. Figure 7 gives an example of imperfect segmented cine, while the real-time retrospective reconstruction shows good image quality. Here both segmented and real-time cine were acquired at the identical slice location and orientation (Additional file 8: Figure S7.avi).

The quantitative results of image quality scores are listed in Table 1. For both matrix sizes, real-time results had slightly higher scores for short axis images. The differences were significant for the 192 × 128 matrix. For the long axis views, the segmented cine trended toward higher scores, but no significant differences were found for both matrix sizes. While the mean scores from the two reviewers were used for comparison, there were no significant differences between two experts (one-way ANOVA test, P = 0.596). In all cases, the retrospective reconstruction was visually better than the raw real-time in image quality and temporal solution (e.g. Figure 6a vs. Figure 6b and 6c). No attempts were made to quantify the image quality of raw real-time images.

**Table 1:** Summary of image quality scores

**(a) 256 × 144 matrix**				
	**SAX**		**LAX**	
	**SEG**	**Retro RT**	**SEG**	**Retro RT**
Mean	4.52	4.54	4.09	3.99
STD	0.50	0.44	0.70	0.44
P-value	SEG vs. RT: 0.756		SEG vs. RT: 0.475	

**(b) 192 × 128 matrix**				
	**SAX**		**LAX**	
	**SEG**	**Retro RT**	**SEG**	**Retro RT**
Mean	4.31	4.56	4.37	4.29
STD	0.63	0.38	0.30	0.53
P-value	SEG vs. RT: 0.011		SEG vs. RT: 0.463	

*SEG* segmented cine; *Retro RT* retrospective real-time cine.

Paired *t*-test is used. P-value between two experts: 0.435.

**Figure 8:**
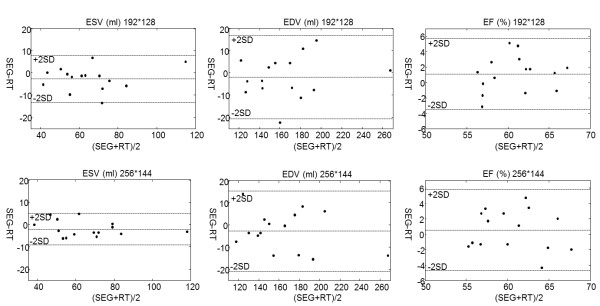
Bland-Altman plots of end-systolic volume and end-diastolic volume measures for segmented and retrospective real-time cine for two acquisition matrix sizes.

Table 2 summarizes the volumetric measurements, showing no significant differences between segmented cine and real-time retrospective reconstruction for EDV and EF (paired *t*-test, P > 0.09). For the ESV, the real-time results showed a trend towards an overestimation on the order of 2–2.5 ml. This difference did not reach statistical significance. The paired *t*-test yielded a p-value of 0.052 and 0.083 for 256 × 144 and 192 × 128 matrix sizes respectively. The volumetric data is compared as Bland-Altman plots in Figure 8. No significant differences were found when comparing measures from the two matrix sizes (paired *t*-test, P > 0.3 for all measures).

**Table 2:** Quantitative measures of cardiac functional parameters

**(a) 256 × 144 matrix**						
	**ESV (ml)**		**EDV (ml)**		**EF (%)**	
	**SEG**	**Retro RT**	**SEG**	**Retro RT**	**SEG**	**Retro RT**
Mean	64.8	66.8	163.8	166.5	60.8	60.3
STD	18.7	19.6	36.0	38.5	3.87	4.20
P-value	SEG vs. RT : 0.052		SEG vs. RT : 0.268		SEG vs. RT : 0.439	

**(b) 192 × 128 matrix**						
	**ESV (ml)**		**EDV (ml)**		**EF (%)**	
	**SEG**	**Retro RT**	**SEG**	**Retro RT**	**SEG**	**Retro RT**
Mean	64.2	66.7	164.9	166.7	61.4	60.3
STD	18.2	17.4	36.5	35.0	3.83	3.42
P-value	SEG vs. RT : 0.083		SEG vs. RT : 0.460		SEG vs. RT : 0.093	

Paired *t*-test is used.

To further illustrate the achieved image quality of retrospective cine reconstruction and highlight the advantage of left ventricular coverage under complete free-breathing, more examples are provided for both imaging matrix sizes (16 s acquisition: Additional file 9: SAX_Stack_192-128_1.avi, Additional file 10: SAX_Stack_192-128_2.avi, Additional file 11: SAX_Stack_192-128_3.avi; 20 s acquisition: Additional file 12: SAX_Stack_256-144_1.avi, Additional file 13: SAX_Stack_256-144_2.avi, Additional file 14: SAX_Stack_256-144_3.avi).

## Discussion

Patients with cardiovascular disease are known to suffer from dyspnea or shortness of breath. The use of breath-held, segmented acquisitions to evaluate cardiac function can be problematic in this group of patients. Alternative real-time, free-breathing imaging techniques have obvious patient comfort and workflow advantages over the breath-held acquisition for subjects who cannot hold their breath well or who suffer from arrhythmias. But the real-time acquisition still cannot provide equivalent spatial and temporal resolution when compared to the segmented cine, even with accelerated imaging technique and fast pulse sequences. To overcome these drawbacks, retrospective reconstruction of real-time cine has been proposed and demonstrated potential to achieve similar image quality as a segmented scan. But previous work required relatively lengthy acquisition of 30-60 s per slice. Furthermore, previous studies have not evaluated whether the volumetric measurements obtained from such retrospective reconstructions are comparable to breath-held, segmented cine acquisitions. This study demonstrated that the acquisitions could be shortened to 16-20 s per slice by employing a non-linear reconstruction to fill ‘holes’ in the re-binned *k*-space data of individual cardiac phases. The technique provides comparable nominal temporal and spatial resolution (1.3-1.8 × 1.8-2.1 mm^2^ pixel size and 30 retro-gated cardiac phases) to a standard breath-held, segmented acquisition. A validation was performed on 15 healthy volunteers with two matrix sizes, and 8–12 short axis slices covering the ventricles in addition to the common long-axis views.

To fill the ‘holes’ in the incomplete binned *k*-space, a non-linear SPIRiT reconstruction was utilized in the proposed solution. Because of the irregular undersampling pattern of binned *k*-space, the standard SENSE or GRAPPA cannot be applied. It should be clarified that this irregular *k*-space is a consequence of temporal binning. To handle this irregularity, iterative SENSE or linear SPIRiT (equation 3 only) may be applicable. The reason for not using iterative SENSE here is its low tolerance to reduced FOVs. With the free-breathing acquisitions, it is not uncommon that part of the chest wall can move out of the FOV leading to severe artifacts in the center of images when using SENSE reconstruction. The linear SPIRiT reconstruction, on the contrary, is robust against fold-over artifacts. But it does not utilize temporal redundancy in the cine images and leads to elevated g-factor. The proposed solution initializes the non-linear reconstruction with the results of linear SPIRiT and often reaches convergence with only a few iterations (<5 in our experiments, the convergence is considered to be reached when the cost function stops decreasing). Although the non-linear conjugate gradient solver may not guarantee to find the global minima, the linear spirit problem (equation 3) can be solved to closely approach its best solution. Experiments show this strategy is robust for the cases tested in this study. As a result, the implemented non-linear temporal SPIRiT consistently gave good image quality for the free-breathing acquisition.

Robustness of proposed method for free-breathing whole ventricle coverage was demonstrated through expert scoring of all images. The real-time retrospective reconstructions had significantly higher image quality scores for the lower resolution matrix size, which was attributed to some cases with breathing artifacts in the segmented acquisitions. The myocardium was successfully segmented in all subjects to obtain volumetric measurements. The study did not find any differences in the volumetric measurements with different matrix sizes as an indication that the improved matrix size does not add to the accuracy of the basic volumetric measurements. The improved resolution may, however, have value when studying regional wall motion. The value of the improved resolution was not studied here, but the results show that the proposed technique can be used to obtain such higher resolution images at the expense of a few extra seconds of scanning and longer reconstruction times.

There is the trade-off between achievable spatial/temporal resolution and acquisition duration of real-time cine. Higher matrix size demands longer acquisition to maintain the achievable resolution and image quality. In this study, the 16 s and 20 s acquisition for two matrix sizes was found to be robust in terms of image quality and provided close agreement with the segmented cine in volumetric measures. The results did however reveal a trend towards a small bias (overestimation) in the ESV on the order of 2–2.5 ml. This bias did not quite reach statistical significance in the present study. An argument could be made that since multiple *t*-test are done, a Bonferoni correction of the significance level should have been employed pushing the observed p-values further away from significance level. The results may also point towards slight temporal blurring. There could be multiple sources of this loss of temporal resolution. When doing the non-rigid motion correction, the proposed method uses a series of images interpolated (in time) from real-time images. The registration could affect the cardiac motion to some small degree in addition to the breathing motion. The proposed method also uses spatio-temporal regularization to reduce noise amplification from the parallel imaging reconstruction. This regularization based gain in signal-to-noise must come at the expense of some small loss in temporal fidelity. The temporal regularization (e.g. ***λ*** in equation 4) could be relaxed at the expense of signal-to-noise, but this was not investigated in detail in this study.

The current study demonstrates that a free-breathing technique can be used to cover the ventricles in a continuous acquisition of 3-4 mins. While this already represents a practical improvement over current practice, the scan duration could conceivably be further reduced using on-the-fly monitoring the filling status of the *k*-space bins for each cardiac phase. When sufficient data has been acquired, the pulse sequence could be notified to stop the scan by real-time feedback from the reconstruction. With such an adaptive approach, the robustness in the presence of irregular respiratory patterns could be further improved and unnecessary acquisition could be avoided. The 16-20 s duration approximates the length of a typical 10-12 s breath-hold plus a pause of 8-10 s. Thus the total acquisition time to cover the myocardium is nominally similar when comparing segmented and free-breathing cine. However the real-time approach should improve patient comfort and simplify the scan operation. In many cases, more time is also added the breath-hold approach when slices need to be repeated due to inadequate breath-hold compliance or when the patient needs more time to recover between breath-holds.

The proposed real-time imaging strategy can avoid ghosting or blurring often seen on the segmented cine due to residual respiratory motion. Compared to the real-time cine without retrospective reconstruction, the proposed approach generates retro-gated images covering the entire cardiac cycle similar to the segmented cine. This eases the utilization of software tools to quantify the global and regional cardiac function, since existing commercial software is mostly designed to handle breath-hold segmented cine images, and are not necessarily optimal for real-time images.

The proposed retrospective reconstruction can be further extended for different arrhythmia cases. The simple retrospective arrhythmia rejection scheme, which is implemented in the present study, may be insufficient to handle some arrhythmic conditions (e.g. severe atrial fibrillation). More effective arrhythmia rejection scheme can be implemented by simultaneously analyzing the recorded ECG waves during the scan and easily combined with proposed reconstruction workflow. While the current study shows the implemented simple arrhythmia rejection strategy is sufficient for normal volunteers, more heartbeats may be discarded for patients with strong irregular cardiac rhythm. This will lead to longer acquisition and also indicates the necessity to implement the above-mentioned adaptive acquisition strategy. The validation of proposed method on patients with known cardiovascular diseases is an important part of our future work.

The proposed algorithm utilizes non-rigid motion correction to eliminate artifacts and blurring from in-plane motion but there is no motion correction for through-plane motion. To minimize the amount of residual through-plane motion, the proposed technique includes a respiratory gating scheme, which discards data acquired during large respiratory excursions. With the properly selected respiratory acceptance window, the non-rigid registration is able to eliminate in-plane motion and non-rigid distortion. However, any residual through-plane motion cannot be corrected in the current 2D acquisition scheme.

As indicated in Figure 4, the retrospective reconstructed real-time cine images often show less flow induced artifacts. The reason is the intrinsic averaging of retrospective reconstruction. The beat-to-beat variation of flow effects can be largely averaged out in the proposed reconstruction with real-time acquisition, while the segmented scan cannot reduce this effect.

**Figure 9:**
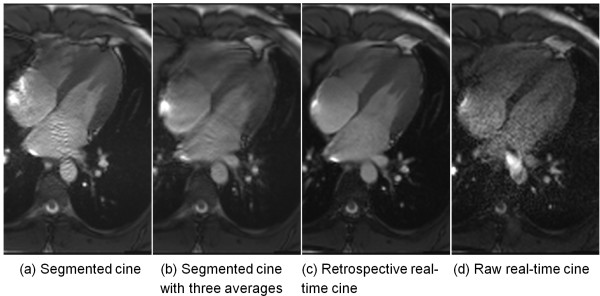
**An example of multiple average segmented cine. (a)** The breath-held segmented cine; **(b)** Segmented cine acquired under normal breathing with three times averaging. **(c)** The retrospective real-time cine reconstructed from 20s acquisition; **(d)** The raw real-time cine with lower temporal resolution and SNR. The acquired matrix size for this case is 192 × 128. The breath-held cine acquisition took 11s and the three times averaged segmented cine took 31 s to acquire.

The presented technique enables improved image quality in patients who are unable to hold their breath. Clinically, these patients are often scanned using multiple averages to eliminate motion artifacts, which can work reasonably well in many patients, but in our experience it lacks the robustness of the presented technique. Figure 9 compares the multiple average segmented cine under free-breathing and retrospective reconstruction (Additional file 15: Figure S9.avi). In this example, the three times averaged segmented cine took 31 s to acquire, while retrospective reconstruction used 20 s of data. Although the reconstruction is much simpler in the multiple averages case, the image quality is worse than the retrospective reconstruction. This multiple average strategy may provide images that are adequate for global cardiac functional assessment, but they may not be good enough for detailed wall motion analysis.

**Figure 10:**
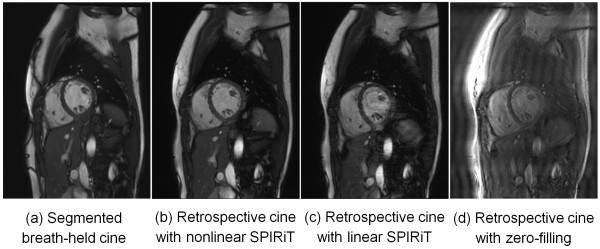
**Comparison of linear reconstruction and non-linear reconstruction for retrospective cine. (a)** Segmented breath-held cine; **(b)** Retrospective cine using the non-linear SPIRiT; **(c)** Retrospective cine using the linear SPIRiT (equation 3). **(d)** Retrospective cine with only zero-filling. No parallel imaging is applied on the binned *k*-space. The insufficient performance of linear SPIRiT is improved by the non-linear regularization.

It was found in this study that the retrospective binning of data into cardiac phases can leave relatively large holes in *k*-space that need to be filled by the reconstruction. A non-linear reconstruction approach was chosen here since it was found to be more robust across the cases studied here. Figure 10 illustrates the benefits of non-linear reconstruction more explicitly (Additional file 16: Figure S10.avi). As illustrated in this example, the image quality of linear reconstruction is significantly better than those without parallel imaging (Figure 10d), but it still can be worse than non-linear method utilizing the temporal redundancy. In practice, the performance of a purely linear method was found to be variable, depending on the size of *k*-space ‘holes’, while the non-linear method gave more robustness with the price of slightly enlarged temporal footprint and a marginally increased reconstruction time. One focus of this study was to shorten the total length of the acquisition and under those conditions the non-linear reconstruction was found to be critical. If a longer acquisition is performed, the ‘holes’ in *k-*space are smaller on average and the linear reconstruction may suffice.

The scanner integration and inline reconstruction of proposed method was achieved using the Gadgetron framework. Multi-threading was intensively used to utilize the full computational power of sixteen cores on the reconstruction computer. However, it still took 80–120 seconds to reconstruct a single slice with the computational hardware used in this study. For example, for a 16s real-time data with 192 × 128 matrix size and 181 acquired raw real-time images, the total reconstruction time is 82.0 s, where motion correction and warping *k*-space took 23.8 s and the conjugate solver took 37.3 s. The rest time was occupied by TGRAPPA reconstruction, coil map estimation, respiratory navigator estimation, etc. For a multi-slice scan to cover the entire heart, the waiting time after the completion of data acquisition (because the reconstruction is partially overlapped with data acquisition) is about 8–12 minutes. This moderate delay time may be worthwhile, because the scan procedure was simplified by eliminating the repeated breath-holding and the ventricles could be completely covered with free-breathing acquisition. On the other hand, the clinical applicability can be improved with further shortened reconstruction. Since every slice is reconstructed independently, it is straightforward to parallelize the reconstruction further by simply allowing the reconstruction software to run on multiple nodes in parallel. Such a multi-node implementation is the subject of future work.

## Conclusions

In this study we have proposed a new retrospective reconstruction scheme for real-time cine imaging with the emphasis on shortening the required acquisition duration by performing non-linear reconstruction. The study shows that a shortened acquisition of 16-20 s can be achieved while producing comparable image quality to a segmented cine with good spatial and temporal resolution. The quantitative measures of cardiac function obtained using the proposed real-time technique show good agreement with the segmented, breath-held technique.

## Abbreviations

CMR: Cardiovascular magnetic resonance; ECG: Electrocardiography; ESV: End-systolic volume; EDV: End-diastolic volume; ACS: Auto-calibration signal; SAX: Short axis; LAX: Long axis; FOV: Field of view.

## Competing interests

Dr. Arai is a principal investigator on a US government Cooperative Research And Development Agreement (CRADA) with Siemens Medical Solutions (HLCR-05-004).

## Authors’ contributions

HX conceived of the study, developed algorithms and software, performed processing and analysis, and drafted the manuscript. PK conceived of the study, developed algorithms and software and performed analysis of image quality. GLR performed the quantitative measurement of cardiac function and performed analysis of image quality. AEA participated in design of the study and performed analysis of image quality. MSH conceived of the study, developed algorithms and software and performed analysis of image quality. All authors read and approved the final manuscript.

## Supplementary Material

Additional file 1: Figure S3a Movie for the Figure 3a, shows the FFT reconstruction is not robust for the shortened acquisition. Click here for file

Additional file 2: Figure S3b Movie for the Figure 3b, shows retrospective reconstruction improves the image quality. Click here for file

Additional file 3: Figure S4 Movie for the Figure 4, gives an example of *k-space* holes after the rebinning. Click here for file

Additional file 4: Figure S5a Movie for the Figure 5a, gives an example of retrospective reconstruction for a two-chamber view. Click here for file

Additional file 5: Figure S5b Movie for the Figure 5b, gives an example of retrospective reconstruction for a four-chamber view. Click here for file

Additional file 6: Figure S5c Movie for the Figure 5c, gives an example of retrospective reconstruction for a three-chamber view. Click here for file

Additional file 7: Figure S6 Movie for the Figure 6, demonstrates the improved visualization of mitral valve of retrospective reconstruction. Click here for file

Additional file 8: Figure S7 Movie for the Figure 7, illustrates a segmented cine with breathing artifacts, while the retrospective reconstruction still gives good image quality. Click here for file

Additional file 9 **SAX_Stack_192-128_1.** An example of multiple SAX views to cover the left ventricle for the 192×128 acquisition matrix. Click here for file

Additional file 10 **SAX_Stack_192-128_2.** An example of multiple SAX views to cover the left ventricle for the 192×128 acquisition matrix. Click here for file

Additional file 11 **SAX_Stack_192-128_3.** An example of multiple SAX views to cover the left ventricle for the 192×128 acquisition matrix. Click here for file

Additional file 12 **SAX_Stack_256-144 1.** An example of multiple SAX views to cover the left ventricle for the 256×144 acquisition matrix. Click here for file

Additional file 13 **SAX_Stack_256-144_2.** An example of multiple SAX views to cover the left ventricle for the 256×144 acquisition matrix. Click here for file

Additional file 14 **Stack_192-144_3.** An example of multiple SAX views to cover the left ventricle for the 256×144 acquisition matrix. Click here for file

Additional file 15: Figure S9 Movie for the Figure 9, shows the retrospective reconstruction can outperform the multiple averaging acquisition strategy. Click here for file

Additional file 16: Figure S10 Movie for the Figure 10, illustrates the image quality improvement of non-linear reconstruction over the linear scheme. Click here for file
